# Synthesis of Mesoporous Alumina with High Specific Surface Area via Reverse Precipitation Method for Enhanced Adsorption and Regeneration of Congo Red

**DOI:** 10.3390/ma18112656

**Published:** 2025-06-05

**Authors:** Shuaiqi Chen, Ziqiang Zhao, Boning Jiang, Yuanchao Zhang, Xuhui Wang, Xiangyu Xu, Jiaqing Song

**Affiliations:** 1 College of Chemistry, Beijing University of Chemical Technology, Beijing 100029, China; 2022430033@mail.buct.edu.cn (S.C.); 13208461283@163.com (Z.Z.); 2023400285@mail.buct.edu.cn (B.J.); 15168977688@163.com (Y.Z.); xuxy@mail.buct.edu.cn (X.X.); 2 State Key Laboratory of Chemical Resource Engineering, Beijing University of Chemical Technology, Beijing 100029, China; 3 Sinopec Catalyst Company Limited, Beijing 100176, China; 2016400154@mail.buct.edu.cn

**Keywords:** mesoporous alumina, high specific surface area, high adsorption capacity, thermal stability, regeneration

## Abstract

Various forms of alumina have attracted considerable attention for their ability to remove anionic dyes from wastewater, attributed to their high specific surface area, and environmental safety. In this study, a series of modified alumina materials were synthesized for the first time using the reverse precipitation method with dual aluminum sources and without template agent to explore their applicability in various scenarios, including adsorption processes and regeneration cycles. The results revealed that non-modified alumina exhibited superior adsorption properties, while silicon-modified alumina demonstrated exceptional thermal stability during high temperature calcination. For silicon-modified alumina, the replacement of some Al–OH groups with silicon resulted in the formation of a protective silicon layer on the alumina surface, which delayed the sintering process. The pseudo-second-order kinetic model and Langmuir model were utilized to fit the experimental data. Furthermore, the adsorption and regeneration properties of silicon-modified alumina were investigated, revealing a maximum equilibrium adsorption capacity of 822.6 mg/g for Congo Red using non-modified alumina. Notably, the non-modified alumina demonstrated a 40.6% increase in its adsorption capacity compared to its initial capacity after six regeneration cycles at 1000 °C.

## 1. Introduction

Water contaminated with various pollutants, including toxic heavy metals and dyes, has detrimental effects on human health [1]. Research has shown that approximately 10,000 dyes with diverse chemical structures are used in industries such as textiles, rubber, paper, plastics, leather, food, cosmetics, and others. It is estimated that annually, 50% of the 800,000 tons of synthetic dyes produced are azo dyes [2,3]. Approximately 2% of these dyes are discharged into ecosystems, causing significant harm to living organisms. Congo Red (CR) is one of the most widely used dyes in the textile industry and exhibits toxicity toward both animals and plants. Thus, its removal from wastewater is of great environmental importance. Numerous technologies have been developed to address the removal of dyes from aqueous solutions, including adsorption, coagulation/flocculation, advanced chemical oxidation, ozonation, reverse osmosis, membrane filtration, and electrochemical techniques [4,5,6,7,8,9,10]. Among these methods, adsorption is widely used as the most convenient and extensively used technique in water treatment. Furthermore, adsorption offers several advantages over alternative methods, including ease of operation, applicability, cost-effectiveness, and reproducibility [11,12]. Various adsorbents, such as activated carbon [13], clay [3], waste materials [14], rice husk [15], and fly ash [16], have been investigated for their efficacy in CR removal.

Alumina (Al_2_O_3_) is widely acknowledged as an environmentally friendly material [17]. The wide application of alumina arises from its high specific surface area, pore volume, and pore size distribution, as well as its acid/base characteristics, which are primarily influenced by surface chemical composition, local microstructure, and phase composition. However, the chemical and hydrothermal stability of γ-Al_2_O_3_ are crucial factors for regeneration and catalytic reactions. At higher reaction temperatures (650–1200 °C) and especially in the presence of water vapor, the porous structure of γ-Al_2_O_3_ collapses due to sintering and structural transformations to other forms of alumina (α-Al_2_O_3_), resulting in lower porosity of the supports and, in some cases, deactivation of the catalyst and adsorbent. Recently, Song et al. [18,19,20,21] synthesized boehmite (AlOOH) and γ-Al_2_O_3_ using NaAlO_2_ and Al_2_(SO_4_)_3_ as raw materials. The results exhibited a higher specific surface area (678.4 m^2^/g) and thermal stability compared to commercial alumina, attributed to their morphology retention. Wu et al. [22] synthesized a series of γ-Al_2_O_3_ modified with varying amounts of polyethylene glycol (PEG) by a reverse precipitation method. The study revealed that 1 mol% PEG-modified γ-Al_2_O_3_ exhibited exceptional thermal stability after calcination at 1100 °C for 3 h, transforming into θ-Al_2_O_3_ with a specific surface area of 92.2 m^2^/g and a pore volume of 0.77 cm^3^/g. Hu et al. [23] prepared mesoporous γ-Al_2_O_3_ through a reverse precipitation method, utilizing Si-doped Al(OH)_3_ and HNO_3_ as raw materials. The prepared γ-Al_2_O_3_ maintained a relatively high specific surface area of 110.0 m^2^/g and a pore volume of 0.73 cm^3^/g at 1100 °C. Al-Salihi et al. [24] synthesized highly porous γ-Al_2_O_3_ nanoshells by applying a novel deposition technique to coat carbon black (CB) with alumina. These nanoshells demonstrated a maximum adsorption capacity of 370.4 mg/g of CR removal from aqueous solutions. Li et al. [25] fabricated mesoporous γ-Al_2_O_3_ nanofibers with high pore volume using a template-free method in a membrane dispersion microreactor, followed by calcination. These fibers exhibited an exceptional adsorption capacity of 1323.7 mg/g for CR. Chen et al. [26] fabricated an ultrathin-walled graphitic mesoporous carbon that demonstrated an experimental maximum adsorption capacity of 738.9 mg/g for CR. Remarkably, the adsorption rate remained almost unchanged even after five regeneration cycles.

Under high pH conditions, conventional precipitation generally leads to the formation of large polynuclear species [AlO_4_Al_12_(OH)_24_(H_2_O)_12_]^7^⁺, whereas reverse precipitation yields smaller polynuclear species Al(OH)_4_^−^ [27]. As a result, the reverse precipitation method produces alumina with a significantly higher specific surface area, owing to the formation of smaller polynuclear species. For the first time, this study developed the reverse precipitation method with dual aluminum sources and without the template agent to synthesize non-modified alumina and silicon-modified alumina. It also analyzed the main factors influencing the specific surface area and thermal stability of alumina. First, we discussed the significance of the synthesis route, including the incorporation of silicon, followed by the impact of high-temperature calcination on adsorbent stability. Subsequently, we investigated the adsorption processes and regeneration cycles of alumina for CR removal.

## 2. Materials and Methods

### 2.1. Materials

Sodium hydroxide (AR), aluminum hydroxide (AR), and aluminum sulfate octadecahydrate (AR) were obtained from Xilong Scientific Company Limited. (Shantou, China). Water glass solution (SiO_2_ 27.4 m%) was sourced from Sinopec Catalyst Company Limited. (Beijing, China). Na_2_HPO_4_·12H_2_O (AR) was acquired from Beijing Chemical Works (Beijing, China). Congo Red (≥97.0%, HPLC) was acquired from Sigma Aldrich (Shanghai, China) Trading Company Limited. Sasol boehmite (SB) was sourced from Sasol Germany GmbH (Hamburg, Germany).

### 2.2. Synthesis

The sodium metaaluminate solution: 0.5640 mol (22.56 g) of NaOH was added to 0.1258 mol (9.82 g) of Al(OH)_3_ and 67.62 mL H_2_O in a 100 mL Teflon-lined stainless-steel autoclave sealed and heated at 160 °C for 4 h.

The aluminum sulfate solution: 0.2 mol (133.2 g) of Al_2_(SO_4_)_3_·18H_2_O was added to 866.8 mL of H_2_O in a beaker and stirred at room temperature to complete the dissolution of the solid materials.

The preparation of alumina via the reverse precipitation method typically involves the calcination of the precipitate formed from the reaction of sodium aluminate and aluminum sulfate in an aqueous solution. Initially, Al_2_(SO_4_)_3_ solution was added dropwise to NaAlO_2_ solution at matched rates while under magnetic stirring, resulting in immediate white precipitation. The rates of adding the acid solution and precipitant were controlled to keep the reaction mixture at pH = 8. The resulting precipitate was subjected to crystallization at 120 °C for 3 h in a hydrothermal reactor. The non-modified boehmite (BO1) was then collected by filtration and thoroughly washed with distilled water. In the case of silicon-modified boehmite (BO2), sodium silicate was added after the crystallization process, with a silicon mole fraction maintained at 42%, and the product was collected by filtration and thoroughly washed. The samples were prepared as follows: AL1-600 refers to BO1 calcined at 600 °C for 2 h, AL1-1000 refers to BO1 calcined at 1000 °C for 2 h, AL2-600 refers to BO2 calcined at 600 °C for 2 h, AL2-1000 refers to BO2 calcined at 1000 °C for 2 h, and SB-600 refers to SB (Sasol boehmite, Brunsbüttel, Germany) calcined at 600 °C for 2 h.

### 2.3. Analytical Procedures

X-ray diffraction analysis was performed utilizing a D8 Advanced Bruker diffractometer (Bruker, Karlsruhe, Germany), equipped with a copper anode and set to 40 kV and 40 mA. The data acquisition encompassed a 2θ range spanning from 10° to 70°, with a goniometer scanning speed of 5° per minute. The specific surface area and pore size measurements of the sample were obtained through nitrogen adsorption analysis at 77 K, conducted on a TriStar II 3020 apparatus (Micromeritics, Norcross, GA, USA). The specific surface area was subsequently determined using the Brunauer–Emmett–Teller (BET) calculation method. For microstructural examination, a JEM-2100 high-resolution transmission electron microscope (JEOL, Tokyo, Japan) was employed. Thermogravimetric analysis was executed on an STA449F5 Jupiter instrument (Netzsch, Selb, Germany), with a heating rate of 10 °C per minute. Fourier Transform Infrared (FTIR) spectroscopy was carried out using a Nicolet 6700 spectrophotometer from Thermo Fisher (Waltham, MA, USA). Zeta potential measurements were conducted with a ZEN3600 Malvern instrument (Malvern, Marvin City, UK). A minimal quantity of sample was dissolved in potassium nitrate solution, and the pH was fine-tuned using potassium hydroxide and nitric acid prior to measuring the zeta potential of the resultant solution.

### 2.4. Adsorption Studies

Adsorption experiments were performed in batches at ambient temperature to ascertain the adsorption capacity of CR onto the sample material. After completing the adsorption process, the solution, which still contained residual CR, was filtered and subsequently analyzed using a Shimadzu UV-2600 ultraviolet spectrophotometer (Shimadzu, Kyoto, Japan). The equilibrium adsorption capacity (*q_e_*, mg/g) and the adsorption capacity (*q_t_*, mg/g) were calculated according to the following equations (Equations (1) and (2)):

(1) $$q_{e}=\frac{\left(C_{0}-C_{e}\right)\times V}{m}$$

(2) $$q_{t}=\frac{\left(C_{0}-C_{t}\right)\times V}{m}$$

where *C*_0_ (mg/L) represents the initial concentration of CR, *C_e_* (mg/L) is the equilibrium concentration of CR, *C_t_* (mg/L) denotes the concentration of CR at time t (min), V (L) is the total volume of the solution, and m (mg) denotes the mass of the adsorbent.

#### 2.4.1. Adsorption Kinetics and Isotherm

To investigate the impact of the initial CR solution concentrations on adsorption performance, a series of experiments were conducted. Specifically, the adsorption experiments were performed at a stirring speed of 400 rpm, with an adsorbent dose of 0.1 g/L. The CR solution was initially prepared at a concentration of 600 mg/L and then diluted to achieve concentrations of 500, 400, 300, and 200 mg/L, respectively. The experimental data obtained were subsequently fitted to the Langmuir, Freundlich, Temkin, and Dubinin–Radushkevich isothermal adsorption models, which are described by the corresponding equations (Equations (3)–(7)).

(3) $$\frac{C_{e}}{q_{e}}=\frac{1}{{K_{L}q}_{m}}+\frac{C_{e}}{q_{m}}$$

(4) $$lgq_{e}=lgK_{F}+\frac{1}{n}{lgC}_{e}$$

(5) $$q_{e}=\frac{RT}{b_{T}}lnK_{T}+\frac{RT}{b_{T}}{lnC}_{e}$$

(6) $${Inq}_{e}=lnq_{m}+K_{D}\varepsilon^{2}$$

(7) $$E=1/\sqrt{2K_{D}}$$

where *q_m_* (mg/g) represents the theoretical adsorbing capacity of CR, K_L_ is the Langmuir constant, K_F_ is the Freundlich constant, R is the gas constant (8.314 J/mol. K), T (K) is the Kelvin constant, b_T_ (J/mol) is the Temkin constant, K_T_ (L/g) is the Temkin isotherm constant, K_D_ is D–R constant (mol^2^/kJ^2^), and E is the free energy of adsorption (kJ/mol).

The concentration of the CR solution was set at 400 mg/L, and the adsorbent dosage was fixed at 0.1 g/L. The mixture was agitated in a magnetic stirrer at 400 rpm, and the adsorption process was allowed to proceed for various time intervals (5, 10, 20, 30, 60, 120, 240, and 360 min). The experimental data obtained from these adsorption tests were subjected to linear fitting using pseudo-first-order, pseudo-second-order, Elovich, and intraparticle diffusion models, which are described by the corresponding equations (Equations (8)–(11)):

(8) $$\mathrm{lg}\left(q_{e}-q_{t}\right)=lgq_{e}-\frac{k_{1}}{2.303}t$$

(9) $$\frac{t}{q_{t}}=\frac{1}{k_{2}{q_{e}}^{2}}+\frac{1}{q_{e}}t$$

(10) $$q_{t}=A+Blnt$$

(11) $$q_{t}=K_{t}t^{1/2}+C$$

where k_1_ (min^−1^) represents the rate constant of adsorption in the pseudo-first order model, k_2_ (g/mg∙min) denotes the rate constant of adsorption in the pseudo-second order model, A and B are Elovich constants, K_t_ (g/mg·min^−1/2^) is the intraparticle diffusion rate constant, and C is a constant that relates to the boundary layer thickness.

#### 2.4.2. Regeneration Properties of the Adsorbent

To investigate the regeneration characteristics of the adsorbent, an adsorption regeneration experiment was conducted following a calcination process at 600 °C for 2 h.

## 3. Results and Discussion

### 3.1. Characterization of Adsorbents

**Figure 1:**
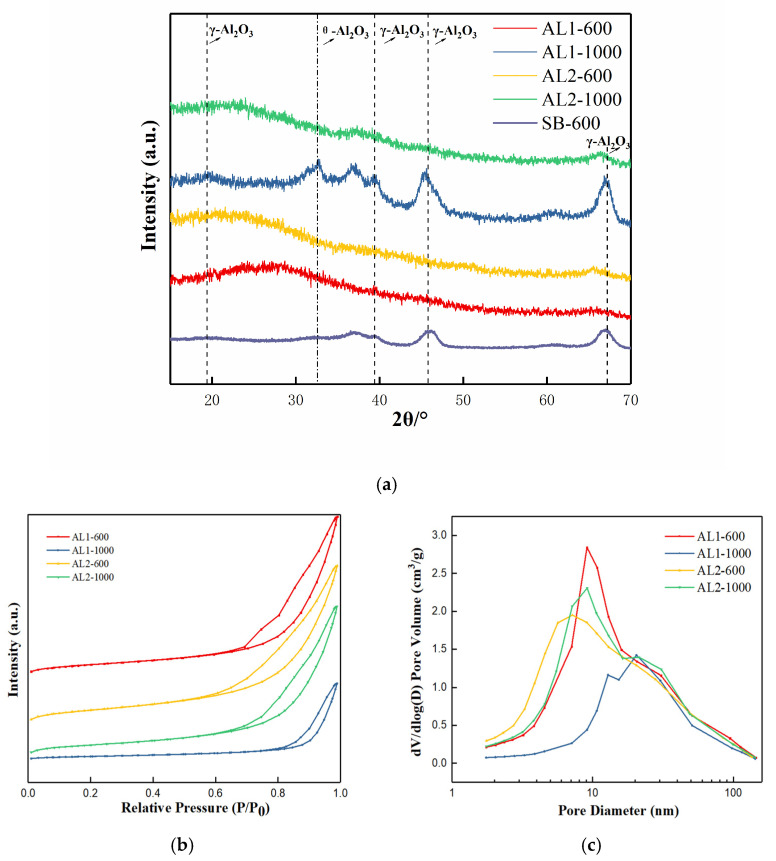
(**a**) XRD patterns. (**b**) N_2_ adsorption–desorption isotherms. (**c**) Pore size distribution of samples.

AL1 and AL2 denote non-modified alumina and silicon-modified alumina, respectively. SB-600 was the sample SB (Sasol boehmite) calcined at 600 °C for 2 h. Figure 1a illustrates the characteristic peaks of γ-Al_2_O_3_ for AL1-600 and AL2-600, which were observed at 2θ values of 39.4° and 67.2°, corresponding to the (2 2 2) and (4 4 0) crystal plane reflections of γ-Al_2_O_3_ (JCPDS No. 10-0425) [22]. After calcination at 1000 °C, AL1-1000 showed transformation to the θ-Al_2_O_3_ phase (JCPDS, No. 11-0517) [20], demonstrating the occurrence of crystalline transformation. However, a significant observation was that AL2-1000 did not undergo this transformation to the θ-phase even under such harsh calcination conditions in air. This suggested a significant improvement in the thermal stability of silicon-modified alumina.

**Figure 2:**
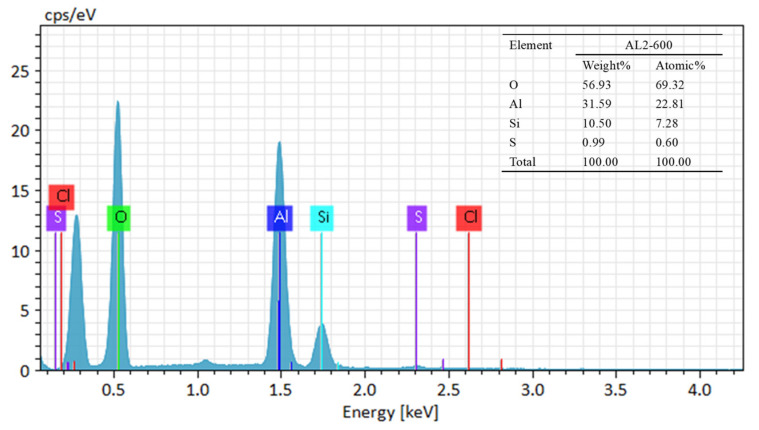
Elemental EDS of AL2-600.

**Figure 3:**
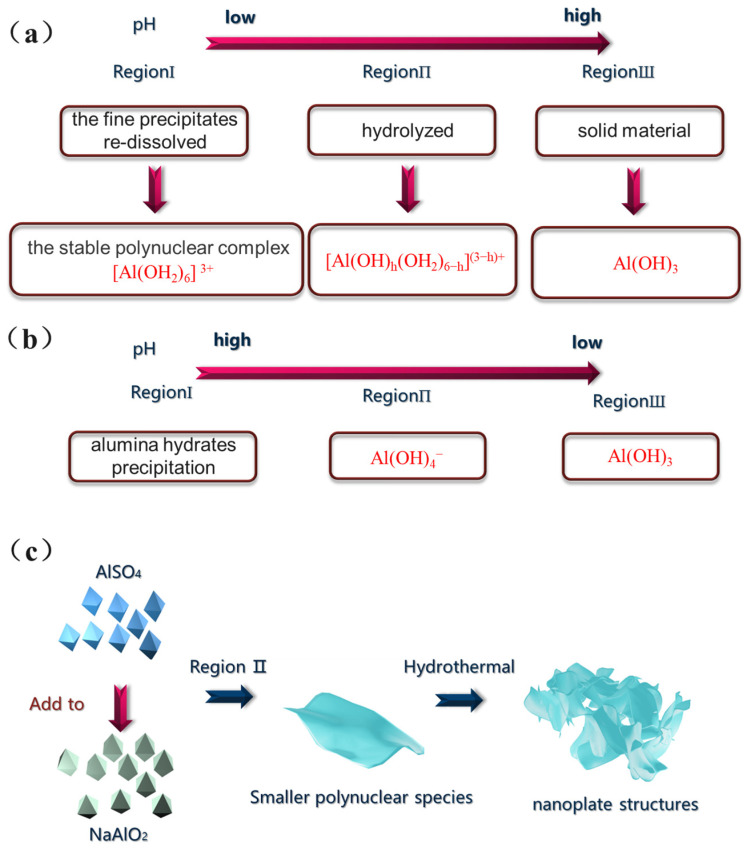
A schematic illustration depicting (**a**) the progression of conventional precipitation, (**b**) reverse precipitation, and (**c**) a diagram of alumina synthesized by the reverse precipitation method.

The sample exhibited a type IV adsorption/desorption isotherm, indicating a mesoporous structure, as illustrated in Figure 1b. As shown in Figure 1c, AL1-600 displayed a relatively concentrated pore size distribution, with a most probable pore size of 10 nm. In contrast, AL2-600 exhibited a most probable pore size of 6 nm, with a relatively broad distribution. After calcination at 1000 °C, the most probable pore size of AL2-1000 was 8 nm, and the most probable pore size of AL1-1000 was 20 nm. As summarized in Table 1, the specific surface areas and pore volumes of AL1 and AL2 exceeded those of SB-600. Specifically, the specific surface area of AL1-600 attained 540.5 m^2^/g but decreased to 173.5 m^2^/g after calcination at 1000 °C. In contrast, the specific surface area of AL2-1000 remained at 496.0 m^2^/g, indicating its superior thermal stability compared to others [20,22,25]. It can also be observed from the EDS characterization in Figure 2 that silicon has been successfully loaded onto the modified alumina. The aforementioned phenomena were attributed to the fact that the reverse precipitation method formed smaller nuclei compared to the conventional method, resulting in a larger specific surface area for the synthesized alumina, as illustrated in Figure 3. Additionally, doping with Si enhanced the thermal stability of alumina.

**Figure 4:**
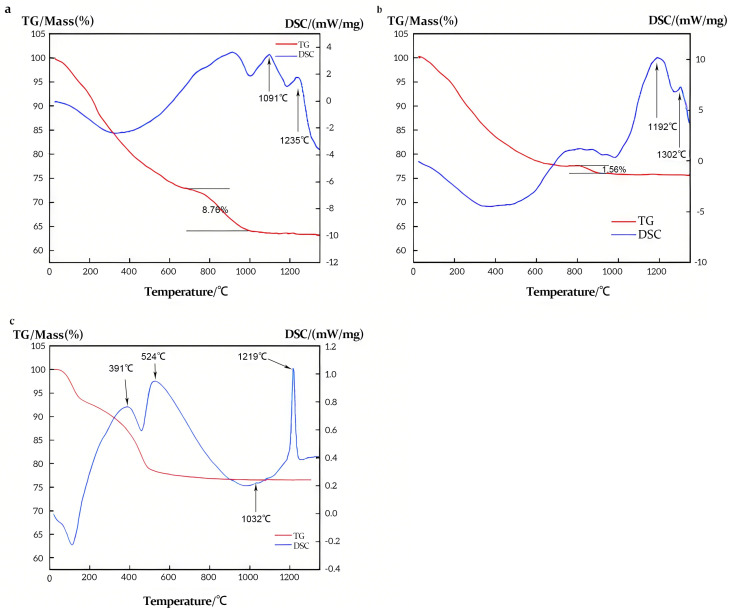
TG-DSC analysis of samples ((**a**): BO1, (**b**): BO2, (**c**): Sasol boehmite).

**Figure 5:**
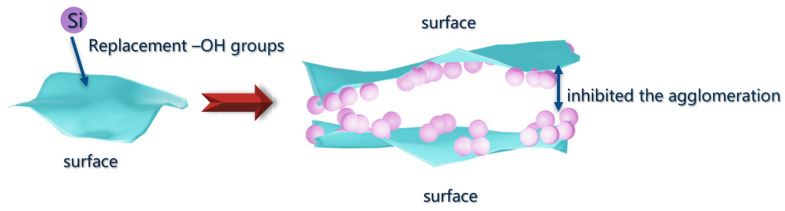
Schematic diagram of the formation a protective silicon layer on the alumina surface.

BO1 and BO2 represented non-modified boehmite and silicon-modified boehmite, respectively. The phase transformation route in the range of 200–1400 °C was as follows: γ-AlOOH → γ-Al_2_O_3_ → θ-Al_2_O_3_ → α-Al_2_O_3_ [27]. An exothermic peak observed at approximately 1091 and 1235 °C is likely attributed to the γ-Al_2_O_3_ to θ-Al_2_O_3_ and θ-Al_2_O_3_ to α-Al_2_O_3_ transformations for BO1, as shown in Figure 4a. The corresponding exothermic peaks for SB materials are 1032 and 1219 °C, as shown in Figure 4c. However, for BO2, the corresponding endothermic peaks for these phase transitions shifted to 1192 °C and 1302 °C, as shown in Figure 4b. Simultaneously, during the dehydroxylation process after 600 °C, the weight loss observed for AL2 was markedly lower, totaling only 1.56%, in contrast to the 8.76% weight loss for AL1. These results demonstrate that the incorporation of silicon significantly alters the phase transformation temperatures of Al_2_O_3_. This phenomenon was attributed to the formation of a protective silicon layer on the alumina surface during the dehydroxylation stage, arising from the replacement of some Al–OH groups with silicon. Consequently, the sintering process was delayed, as illustrated in Figure 5.

**Figure 6:**
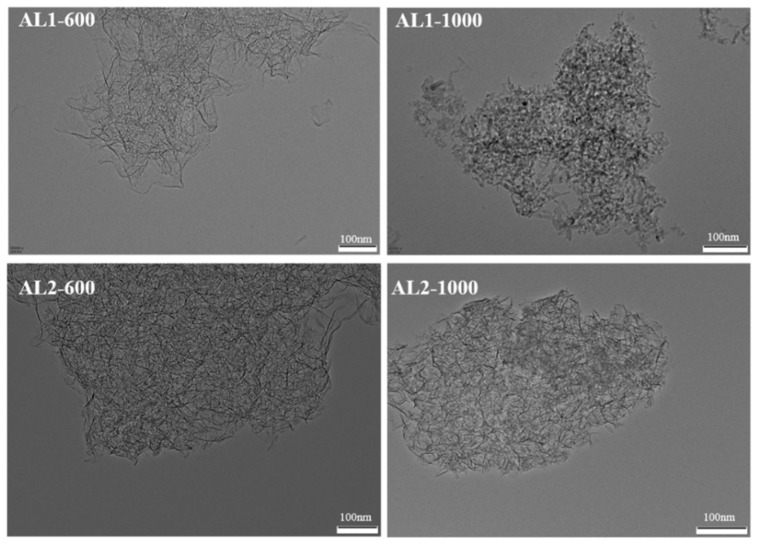
HRTEM images of samples.

Figure 6 shows the HRTEM images of the alumina samples. AL1-600, AL2-600, and AL2-1000 were nanoplate structures, while AL1-1000 exhibited an irregular granular morphology. This was due to significant sintering of AL1 at 1000 °C, whereas AL2 remained almost unsintered at the same temperature. This observation indicates that AL2 possesses superior thermal stability at high temperatures compared to AL1.

### 3.2. Adsorption Behavior

#### 3.2.1. Kinetics of Adsorption

**Figure 7:**
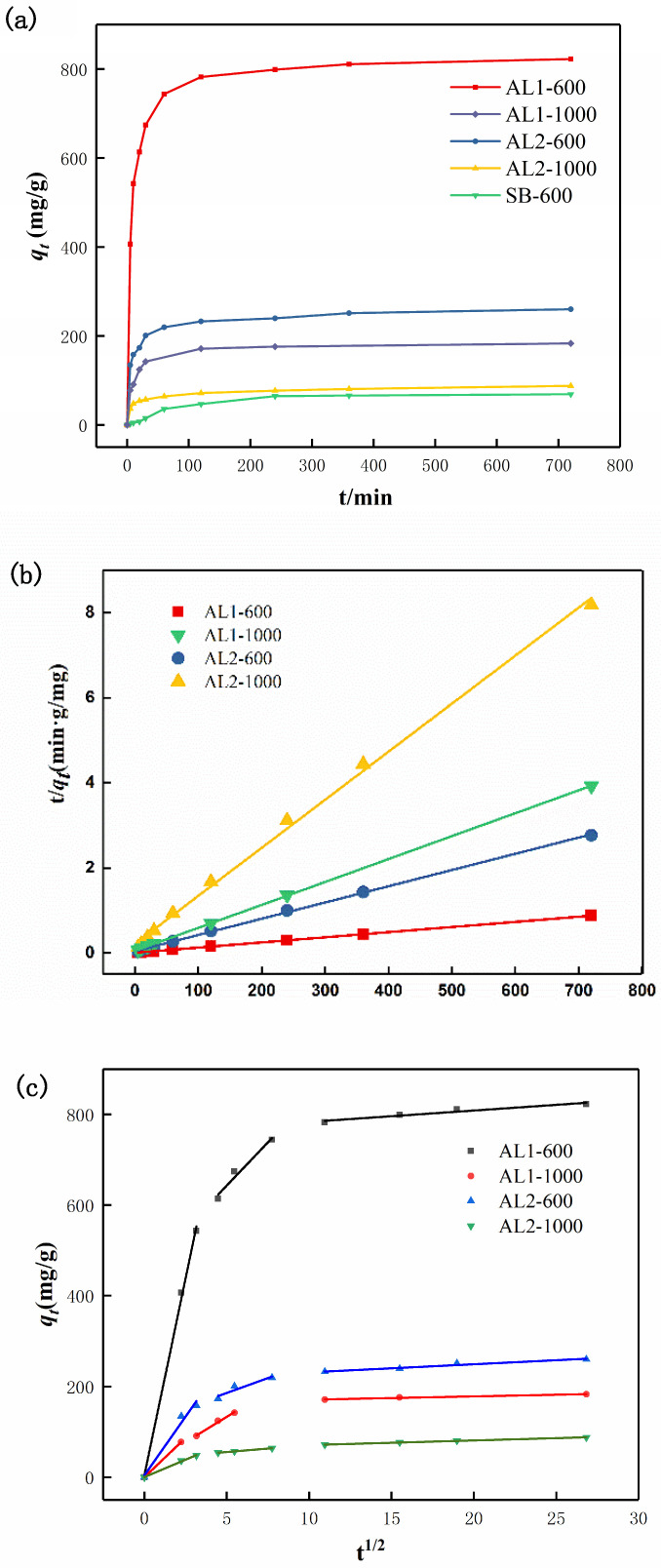
(**a**) Adsorption capacity–time curve, (**b**) pseudo-second-order kinetics, and (**c**) intraparticle diffusion results of the adsorption process of samples to CR.

The adsorption capacity–time curve of CR was investigated, with an initial CR concentration of 400 mg/L and an adsorbent concentration of 0.1 g/L. As shown in Figure 7a, SB-600 exhibited the lowest adsorption capacity for CR, reaching a maximum of only 69.9 mg/g within 360 min. Due to its limited adsorption capacity, SB-600 will not be further discussed. Conversely, AL1-600 demonstrated the highest adsorption capacity for CR, achieving 822.6 mg/g. However, with the introduction of silicon, the adsorption capacity of AL2-600 declined significantly to 260.3 mg/g. In accordance with the specific surface area data in Table 1, AL2-600, with its higher specific surface area, exhibited lower adsorption capacity compared to AL1-600. This discrepancy is attributed to the introduction of silicon, which occupied the active sites on the surface of alumina.

To investigate the adsorption process of alumina on CR, linear kinetic fitting was conducted using the data obtained from the aforementioned adsorption tests. The results of this fitting are demonstrated in Figure 7b,c. As shown in Table 2, the equilibrium adsorption capacities of AL1 and AL2 were found to be more consistent with the results predicted by the pseudo-second-order kinetic model, as evidenced by the R^2^ value approaching 1. This finding revealed that the pseudo-second-order model can describe the chemisorption between adsorbents and CR [28]. As shown in Table 3, the intraparticle diffusion model revealed that there are three potential mechanism stages: (1) the external mass transfer (*K_t1_*); (2) the intraparticle diffusion inside adsorbents (*K_t2_*); and (3) the equilibrium state when intraparticle diffusion slows down due to saturation (*K_t3_*). The rate constant followed the order *K_t1_* > *K_t2_* > *K_t3_*, which confirmed that the external diffusion was faster than intraparticle diffusion and the equilibrium stage.

#### 3.2.2. Adsorption Isotherm Models

**Figure 8:**
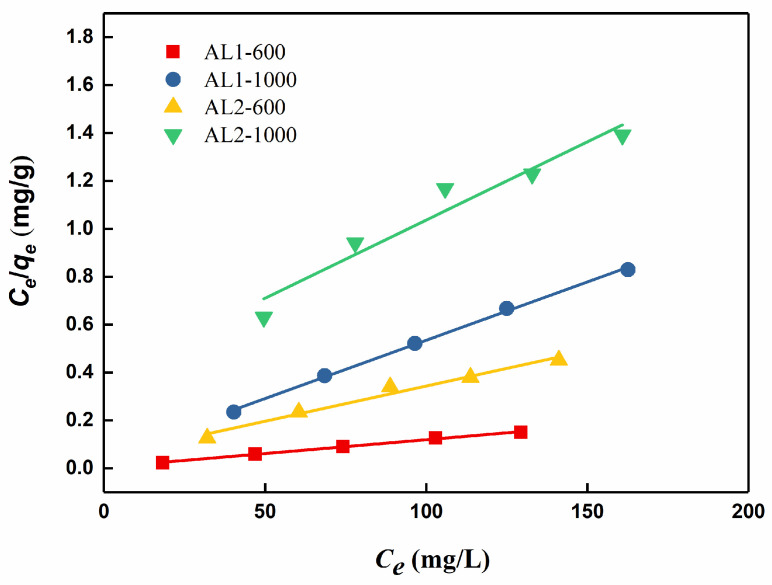
Langmuir fitting of the adsorption of CR by samples.

The adsorption equilibrium mentioned above was analyzed using Four adsorption isotherm models: Langmuir, Freundlich, Temkin and Dubinin–Radushkevich models. The corresponding fits to these models were depicted in Figure 8. Langmuir exhibited higher correlation coefficients compared to the Temkin, Freundlich, and Dubinin–Radushkevich models, as shown in Table 4 and Table 5. This could mean the main mechanisms are chemisorption processes [29].

#### 3.2.3. Adsorption Mechanism

**Figure 9:**
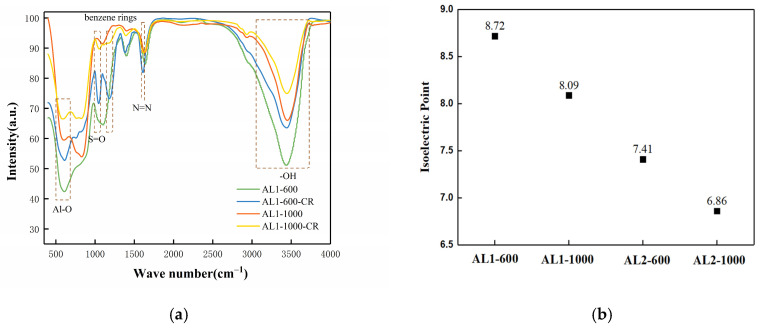
(**a**) FTIR spectra. (**b**) Isoelectric point of samples.

**Figure 10:**
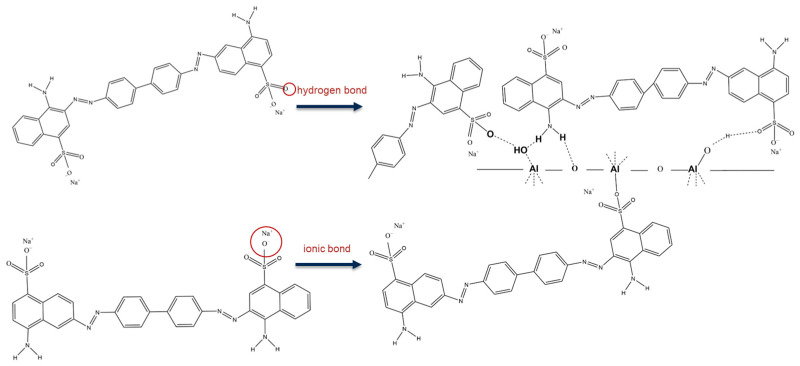
A schematic illustration of the adsorption mechanism for the adsorption of CR onto samples.

As depicted in Figure 9a, AL1-600-CR is denoted as AL1-600 after adsorption. AL1-600 and AL1-600-CR showed peaks at 1046 and 1160 cm^−1^, which are assigned to the stretching vibration of S=O and benzene rings, respectively [24]. The broad band centered at 1604 cm^−1^ refers to the stretching vibration of the N=N stretching of the azo group. Notably, the peak intensity corresponding to the hydroxyl group at approximately 3400 cm^−1^ in AL1-600 was markedly stronger than that observed in AL1-600-CR. Furthermore, the attenuation of stretching vibration peaks at approximately 600 and 800 cm^−1^ suggests the involvement of hydroxyl groups on Si-O and Al-O in the adsorption process. This trend was also observed for AL2-600 and AL2-1000. Additionally, as shown in Figure 9b, AL1-600 possessed the highest isoelectric point among all the samples. The isoelectric point of alumina is highly dependent on the surface hydroxyl groups [30]. A high isoelectric point indicates many hydroxyl groups, resulting in more active sites on the surface. When γ-Al_2_O_3_ is dissolved in water, it forms surface hydroxyl groups that promote the chemical interactions of CR to Al_2_O_3_ [31]. According to the test results and characterization analysis, the possible adsorption mechanism of the adsorbent for pollutants is shown in Figure 10.

Based on the aforementioned adsorption, the kinetics of the adsorption mechanism, and the experimental data, it can be concluded that AL1 provided a greater number of active sites for initial adsorption, while AL2 exhibits superior thermal stability during regeneration.

### 3.3. Recyclability

**Figure 11:**
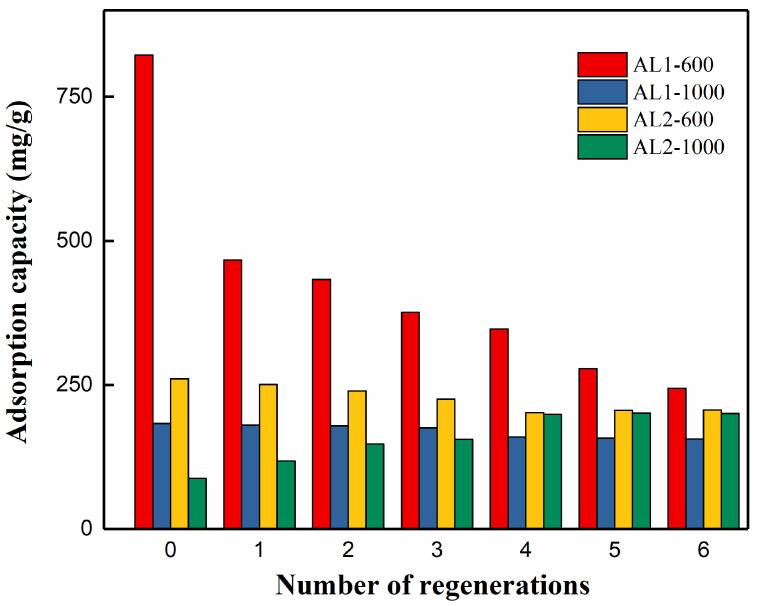
The relationship between the number of adsorbent regenerations and the adsorption capacity.

Due to its significance in cost reduction for practical applications, the recyclability of adsorbents was thoroughly investigated. The adsorption capacity of regenerated samples was compared with that of the pristine sample, as shown in Figure 11 and Table 6 and Table 7. AL1-600 exhibited the most pronounced reduction in specific surface area after six regeneration cycles, decreasing from 540.5 to 303.8 m^2^/g (a 43.8% decrease), with a corresponding adsorption capacity reduction to 244.4 mg/g (a 70.2% decrease). Conversely, AL2-600 demonstrated a 22.6% decrease in specific surface area after six cycles, from 686.2 to 530.8 m^2^/g, accompanied by a 20.7% decrease in adsorption capacity, from 183.3 to 156.0 mg/g. This reduction in adsorption capacity aligns with the decrease in specific surface area. These findings suggested that high-temperature regeneration significantly impacted both the specific surface area and adsorption capacity of AL1-600. Notably, the decline in adsorption capacity was substantially greater than the reduction in specific surface area, indicating that the adsorption sites on the surface were also adversely affected by the regeneration process. After silicon modification, the adsorption capacity of AL2-600 remained relatively stable, with only a slight decrease observed after multiple regeneration cycles at 600 °C.

After undergoing multiple regeneration cycles at 600 °C, the specific surface area of AL2-1000 exhibited only a minimal variation, whereas its adsorption capacity increased significantly, rising from 88.0 to 200.4 mg/g. This can be attributed to the fact that, during the high-temperature regeneration process, silicon migrated from the surface into the interior of AL2-1000. The process exposed more aluminum to the surface, leading to an increase in active sites. A comparison between AL1-1000 and AL2-1000 revealed that the incorporation of silicon had a beneficial effect on the regeneration process.

### 3.4. Comparison with Other Adsorbents

Table 8 presents the adsorption capacity data obtained after researchers selected alumina for the adsorption of CR.

## 4. Conclusions

A series of alumina forms were synthesized by utilizing the reverse precipitation method, resulting in a significant enhancement of their specific surface area. The silicon-modified alumina achieved a specific surface area of 686.2 m^2^/g, whereas the non-modified alumina attained 540.5 m^2^/g. Both silicon-modified alumina and non-modified alumina are much higher than alumina from SB at 600 °C. Additionally, the incorporation of silicon during the synthesis process led to the formation of a protective silicon layer on the alumina surface, thereby enhancing the thermal stability of the alumina and yielding exceptional performance during regeneration. Our results indicated that both non-modified alumina AL1 and silicon-modified alumina AL2 samples exhibited effective adsorption capabilities for CR. AL1-600 exhibited an adsorption capacity as high as 822.6 mg/g, but the adsorption capacity reduced to 244.4 mg/g (a 70.2% decrease) with the sixth regeneration at 600 °C. The adsorption capacity of AL2-600 exhibited a slight decline, whereas the adsorption capacity of AL2-1000 underwent an increase, rising from 88.0 to 200.4 mg/g, after undergoing several cycles of regeneration at 600 °C. The adsorption kinetics and isotherms of these samples for CR were investigated. These results indicated that both AL1 and AL2 exhibited considerable potential as adsorbents for the effective removal of CR from water in diverse scenarios.

## Figures and Tables

**Table 1:** Date table of specific surface area and pore volume of samples.

	BET Surface Area (m^2^/g)	BET Pore Volume (cm^3^/g)
AL1-600	540.5	2.41
AL1-1000	173.5	1.15
AL2-600	686.2	2.33
AL2-1000	496.0	2.25
SB-600	180.4	0.53

**Table 2:** A pseudo-first-order and pseudo-second-order kinetic fitting data table of the adsorption process of CR by the sample.

Adsorbent	*q_t_*-6 h (mg/g)	Pseudo-First-Order			Pseudo-Second-Order		
		*q_e_* (mg/g)	k_1_ (min^−1^)	R^2^	*q_e_* (mg/g)	k_2_ (g/(mg∙min))	R^2^
AL1-600	822.6	228.8	9.189 × 10^−4^	0.95529	826.4	1.803 × 10^−4^	0.99997
AL1-1000	183.4	78.5	0.01119	0.93887	185.5	5.609 × 10^−4^	0.99879
AL2-600	260.3	88.3	0.006633	0.93092	261.8	3.454 × 10^−5^	0.99995
AL2-1000	306.4	38.3	0.005090	0.92107	88.6	6.016 × 10^−4^	0.99941

**Table 3:** An Elovich and intraparticle diffusion fitting data table of the adsorption process of CR by the sample.

Adsorbent	Elovich			Intraparticle Diffusion					
	A	B	R^2^	*K_t1_* (g/mg·min^−1/2^)	R^2^	*K_t2_* (g/mg·min^−1/2^)	R^2^	*K_t3_* (g/mg·min^−1/2^)	R^2^
AL1-600	358.82	79.86	0.89094	173.45	0.99896	38.17	0.96825	2.50	0.94357
AL1-1000	51.15	22.43	0.91613	34.90	0.99999	22.32	0.99035	0.73	0.96650
AL2-600	102.51	25.53	0.96166	51.88	0.97742	13.14	0.89520	1.78	0.95641
AL2-1000	23.51	9.85	0.99423	15.37	0.99579	3.08	0.99999	1.02	0.99092

**Table 4:** A Langmuir and Freundlich fitting data table of adsorption to CR.

	Langmuir			Freundlich		
	*q_m_* (mg/g)	K_L_ (L/mg)	R^2^	K_F_	1/n	R^2^
AL1-600	869.5	0.2584	0.99734	684.2	0.0414	0.70632
AL1-1000	205.3	0.01009	0.99830	151.1	0.1385	0.71412
AL2-600	340.1	0.05909	0.97875	120.5	0.0934	0.96141
AL2-1000	153.1	0.01701	0.94443	19.8	0.3406	0.89287

**Table 5:** A Temkin and Dubinin–Radushkevich fitting data table of adsorption to CR.

	Temkin			Dubinin–Radushkevich			
	*b_T_* (J/mol)	K_T_ (L/mg)	R^2^	*q_m_* (mg/g)	K_D_ (mol^2^/kJ^2^)	E (kJ/mol)	R^2^
AL1-600	73.51	4.72·10^8^	0.69493	822.8	0.000003	408.2	0.37262
AL1-1000	145.22	525.59	0.954529	190.5	0.000032	125.0	0.75629
AL2-600	63.85	16.27	0.70575	289.6	0.000027	136.1	0.40977
AL2-1000	76.60	5.14	0.87057	107.9	0.000150	57.7	0.65687

**Table 6:** Data sheet of BET and adsorption capacity before and after adsorbent regeneration of AL1.

Number of Regenerations	AL1-600			AL1-1000		
	Adsorption Capacity (mg/g)	Specific Surface Area (m^2^/g)	Pore Volume (cm^3^/g)	Adsorption Capacity (mg/g)	Specific Surface Area (m^2^/g)	Pore Volume (cm^3^/g)
0	822.6	540.5	2.41	183.3	161.7	1.09
1	466.7	451.8	1.76	180.0	158.6	1.09
2	432.9	--	--	179.0	--	--
3	376.1	348.0	1.38	175.6	162.9	1.06
4	347.2	--	--	159.7	--	--
5	278.1	--	--	157.9	--	--
6	244.4	303.8	1.25	156.0	152.1	1.05

**Table 7:** Data sheet of BET and adsorption capacity before and after adsorbent regeneration of AL2.

Number of Regenerations	AL2-600			AL2-1000		
	Adsorption Capacity (mg/g)	Specific Surface Area (m^2^/g)	Pore Volume (cm^3^/g)	Adsorption Capacity (mg/g)	Specific Surface Area (m^2^/g)	Pore Volume (cm^3^/g)
0	260.3	686.2	2.33	88.0	496.0	2.25
1	250.7	593.7	2.22	118.2	452.2	2.01
2	239.8	--	--	147.5	--	--
3	225.6	575.4	2.31	155.8	464.5	2.04
4	202.2	--	--	199.0	--	--
5	205.8	--	--	201.1	--	--
6	206.3	530.8	2.20	200.4	477.3	2.11

**Table 8:** Comparison of adsorption capacity of CR removal by different adsorbents.

Adsorbent	Adsorption Capacity mg/g	References
highly porous γ-Al_2_O_3_ nanoshells	370.4	[24]
mesoporous γ-Al_2_O_3_ nanofibers	1323.7	[25]
ultrathin-walled graphitic mesoporous carbon	738.9	[26]
γ-Al_2_O_3_	416.1	[32]
alumina/zirconia composites	60.0	[33]
alumina microspheres	1000.0	[34]
magnetic biochar (MBC1:1)	172.9	[35]
HPMA nanopowder	492.19 (at concentration of 250 mg/L)	[36]
non-modified alumina	822.6	This study
silicon-modified alumina	260.3	This study

## Data Availability

The original contributions presented in this study are included in the article. Further inquiries can be directed to the corresponding author.
